# Do Flower Color and Floral Scent of *Silene* Species affect Host Preference of *Hadena bicruris*, a Seed-Eating Pollinator, under Field Conditions?

**DOI:** 10.1371/journal.pone.0098755

**Published:** 2014-06-06

**Authors:** Paul Page, Adrien Favre, Florian P. Schiestl, Sophie Karrenberg

**Affiliations:** 1 ETH Zurich, Institute of Integrative Biology (IBZ), Zurich, Switzerland; 2 Department of Molecular Evolution and Systematics of Plants, Institute of Biology, University of Leipzig, Leipzig, Germany; 3 Institute of Systematic Botany, University of Zurich, Zurich, Switzerland; 4 Department of Ecology and Genetics, Uppsala University, Uppsala, Sweden; University of Massachusetts, United States of America

## Abstract

Specialization in plant–insect interactions is an important driver of evolutionary divergence; yet, plant traits mediating such interactions are poorly understood. In this study, we investigated how flower color and floral scent are related to seed predation by a seed-eating pollinator. We used field-transplanted recombinant F_2_ hybrids between *Silene latifolia* and *S. dioica* that are the preferred and alternative hosts of the moth *Hadena bicruris* and crosses within these species for comparison. We scored seed predation and flower color and analyzed floral scent. Pinker *S. dioica*-like flowers and emission of α-pinene decreased the odds of seed predation while emission of benzyl acetate and 6-methyl-5-hepten-2-one increased the odds of seed predation. Emission of these compounds did not differ significantly between the two *Silene* species. Our results suggest that flower color plays an important role in the specific interaction of *H. bicruris* with its preferred host *S. latifolia*. The compounds α-pinene, benzyl acetate and 6-methyl-5-hepten-2-one could represent non-specific deterrents and attractants to ovipositing moths. Alternatively, emission of these compounds could be related to herbivory or pathogen attack and act as a signal for host quality. This would weaken the predictability of the plant's costs and benefits of the interaction and act to maintain an imperfect degree of specialization.

## Introduction

Specialization in plant–insect interactions, for example in plant-pollinator interactions, is an important mechanism driving diversification in both plants and insects [1]–[3]. Some insect species are both pollinators and seed predators or herbivores; they lay eggs on plants they pollinate and their larvae consume seeds or vegetative tissues [4]. Associations between plants and such pollinating seed predators (nursery pollinators) or herbivores range from obligate mutualisms, for example in yuccas and yucca moths, where reproduction of both partners depends on the interaction, to parasitism, where the insect damages the plant while providing little pollination service [4], [5]. In non-obligate associations, unresolved evolutionary conflicts are expected: plants gain from attracting insects for pollination but suffer from seed predation or herbivory [5]. The plants' costs and benefits of interacting with nursery pollinators may further depend on the presence of co-pollinators and other herbivores [5]–[8].

The evolution of specific attractants and deterrents is important for the degree of specialization in interactions between plants and pollinating seed predators or herbivores. Hawk moths (*Manduca* species), for example, are pollinating herbivores of Sacred Datura (*Datura wrightii*) and of wild tobacco (*Nicotiana attenuata*). In these systems, both male and female moths are attracted by flower color and scent while oviposition choices by female moths were based on nectar volume or on volatile compounds specifically perceived by female moths [9]–[12]. Interestingly, pollination benefits for *Nicotiana attenuata* were maximized by the presence of both attractants and deterrents for moths as this increased the number of different plants visited [11]. Such a combination of attractants and deterrents could be common [13], and may also be expected where pollinators damage plants through their larvae. More investigations on floral traits are needed to understand their role in the interactions of plants with pollinating seed predators/herbivores, especially under field conditions.

It is difficult to isolate the effect of individual floral traits on insect behavior using naturally occurring variation. Laboratory experiments, on the other hand, may allow only limited inference on natural populations because environmental conditions, as well as herbivores or pests, can strongly influence floral traits, particularly scent [14]–[17]. In addition, learning can be an important determinant of insect behavior in the field [18]. Approaches that allow floral trait manipulation under field conditions include the use of artificial flowers [9], [19], scent addition [20] or genetic technologies such as blocking the expression of biosynthetic pathways using RNA interference [11]. An increasingly used approach for studies comparing inter-fertile populations or species, is the generation of recombinant experimental hybrids [21], for example second-generation hybrids (F_2_, i.e. crosses among first-generation hybrids). F_2_ hybrids carry recombined parental genomes and exhibit highly variable traits, as well as trait combinations that are not present in the parental lineages. For this reason, such hybrids can be used to break up species-specific trait combinations and investigate the effect of uncorrelated floral traits on insect behavior as has been done for example in *Petunia*, *Hemerocallis* and *Silene* [22]–[24]. In this study, we used field-transplanted F_2_ hybrids of two campion species (*Silene*) to investigate the effect of flower color and floral scent on seed predation by the pollinating seed predator *Hadena bicruris*.

We studied *Silene latifolia* Poiret with white flowers and its sister species *Silene dioica* (L.) Clairv. with pink flowers. The two *Silene* species are visited by generalist pollinators such as bumblebees and hybridize naturally; both are dioecious perennials native to and widespread in Europe [25]–[28]. *Silene latifolia* also forms a strong but non-obligate association with the night active moth *Hadena bicruris* Hufn. (Noctuidae, Lepidoptera), a pollinating seed predator [4]. Flowers of both *S. dioica* and *S. latifolia* are open and scented at night when *H. bicruris* is active ([20] personal observation AF, PP). While *S. dioica* flowers open first during the day and remain open, *S. latifolia* flowers open first during the evening, can close during the first days in hot and dry conditions and remain open thereafter if left un-pollinated ([29], personal observation AF, PP, SK). *Hadena bicruris* females mostly lay a single egg on the ovaries of *Silene* flowers and larvae hatch after 3–4 days [30]–[32]. Larvae of *H. bicruris* fully develop and survive on fruits of both *S. dioica* and *S. latifolia*, but larvae reared on *S. latifolia* fruits gained significantly more weight than those reared on *S. dioica* fruits suggesting that *H. bicruris* has specialized in digesting *S. latifolia* tissues [33]. The moth occurs in nearly all populations of *S. latifolia* throughout its native range and is active precisely during its flowering period [33]–[35]. Indeed, in mixed populations of *S. latifolia* and *S. dioica*, the lowest seed predation rates were observed in the earliest flowering *S. dioica* and in the latest flowering *S. latifolia* suggesting that synchronized flowering and activity times play an important role for the specificity of the *S. latifolia* - *H. bicruris* interaction [33], [36].

In contrast to other seed-eating pollinators, both males and females of *H. bicruris* are efficient pollinators of *S. latifolia* during nectar feeding on both male and female *S. latifolia* and show similar visitation patterns [4], [37], [38]. Nocturnal flower visitation including visits of *H. bicruris* to *S. latifolia* caused only little inter-specific pollen transfer between *S. latifolia* and *S. dioica* as compared to diurnal pollination [25], [39]. However, seed eating larvae of *H. bicruris* inflict substantial damage to *S. latifolia* [30], [34], [36], [40], [41]; in a study in the Netherlands, for example, on average 80% of the individuals and 50% of the seed capsules were infested [35]. Selective abortion of infested fruits as a mechanism for reducing the damage by *H. bicruris* was suggested by Burkhardt *et al.* [30], however, the extent to which fruit abortion or other putative mechanisms reduce damage by *H. bicruris* to *S. latifolia* appears to vary widely between populations and experiments [35], [36], [40], [42].

*Hadena bicruris* shows a strong preference for *S. latifolia* when presented with a choice between this species, other white-flowering species or pink-flowering *S. dioica* [31]–[33]. *Hadena bicruris* females also clearly discriminate against *S. latifolia* flowers that provide reduced resources for their offspring such as male flowers, flowers that already have *H. bicruris* eggs or those infested with anther smut [31], [32], [37], [40], [43]. Laboratory and greenhouse experiments suggest that floral scents, in particular lilac aldehydes and phenylacetaldehyde, are important attractors for *H. bicruris* [20], . Furthermore, population size and flower number were shown to influence oviposition and seed predation of *S. latifolia* by *H. bicruris*, however, these effects varied strongly between years, sites and populations [35]. These studies suggest that floral traits play a decisive role for the evolution and specificity of the *S. latifolia* – *H. bicruris* interaction.

The aim of this study was to investigate the roles of flower color and floral scents for the interaction of *H. bicruris* with its preferred host *S. latifolia*. We used field-transplanted *Silene dioica* x *S. latifolia* F_2_ hybrids that expressed a wide range of recombinant phenotypes, as well as crosses within each species. We recorded primary seed predation by *H. bicruris* to identify floral traits leading to moth attack. Our study questions were: (1) Do flower color or floral scent influence primary seed predation? (2) Are traits that increase seed predation specific to the preferred host of *H. bicruris*, *S. latifolia*, or are they shared by the two *Silene* species? We were able to show that both flower color and floral scent affect seed predation in our study system. Our data suggest that flower color is related to host preference, while the scent compounds associated with seed predation could represent unspecific signals.

## Materials and Methods

### Plant material and transplant site

Crosses were derived from 18 plants of each species, originating from three natural populations of each species in the Swiss Alps (Table S1, compare [26], [49], [50]). For this study, we collected data from field-transplanted female plants of second-generation hybrids (F_2_) between *S. dioica* and *S. latifolia* and from second-generation intra-specific crosses between populations of *S. dioica* and between populations of *S. latifolia* (Table S2). In order to obtain a large, highly variable hybrid population we initially transplanted 20 individuals of each of 36 F_2_ families (see below). For comparison, five individuals of each of 18 families of crosses within *S. dioica* and within *S. latifolia* were transplanted (compare Table S2).

The transplant site was located at the edge of a typical natural *S. latifolia* population [26] near Leuk (Valais, Switzerland 46°19′17″N-7°38′46″E, 978 m a.s.l.) as part of an experiment on ecological selection in *S. latifolia*, *S. dioica* and their hybrids and plants were arranged in a field of approximately 7 by 12 m in a randomized block design [49]. The experimental area was part of a field that we rented from its private owner, Armin Bayard. Our study did not involve sampling of or damage to protected species.

The neighboring natural population of *S. latifolia* was large (>500 individuals within 100 m of the experimental plot), co-flowering with our experimental plants and known to be infested by *H. bicruris* (personal observation AF) providing an ideal situation to investigate interactions of naturally occurring *H. bicruris* with experimental plants.

### Flowering phenology, flower number, flower color, and primary seed predation

**Figure 1:**
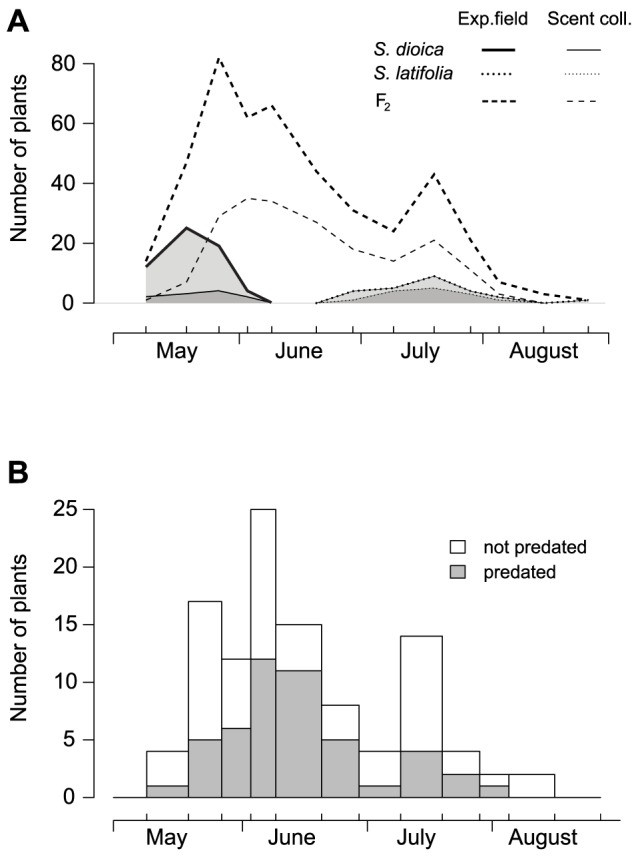
Flowering phenology and seed predation risk. (**a**) Flowering phenology as the total number of female plants in flower in field-transplanted crosses within *Silene dioica*, within *S. latifolia* and in second-generation (F_2_) hybrids between these species; data are presented for the entire experimental field and for a subset of plants used for scent analyses, i.e. those that had newly opening flowers on scent sampling days. (**b**) Seed predation risk over time, expressed as the incidence of seed predation by the moth *Hadena bicruris* in a subset of F_2_ individuals that flowered only for one week such that host choice must have occurred during that week. Days of data collection on flowers are indicated as upward-facing tick marks.

Overall, about two thirds of the experimental plants of *S. dioica*, *S. latifolia* and F_2_ hybrids survived the winter after transplantation and flowered in the following year (2008) where this study was conducted; 48% of the individuals were females (for a detailed account see Table S3). We considered only female plants in this study comprising 31 *S. dioica* (14 families), 33 *S. latifolia* (14 families), and 185 F_2_ hybrids (36 families, compare Table S3). We visited the site every 6 to 11 days during the reproductive phase of the experimental plants (in total 13 times, compare Fig. 1) to score whether or not individuals flowered (at each visit), total flower number (at the end of flowering for each plant), flower color (see below) and seed predation by *H. bicruris* (see below). Each plant was thus checked at all visits. Two *S. dioica* individuals, three *S. latifolia* individuals and six F_2_ individuals could not be scored for seed predation due to herbivory by other animals. We missed flower color for 12 *S. dioica*, five *S. latifolia* and 34 F_2_ individuals because no open flowers were available on the day of scoring (for detailed accounts of survival, flowering and families used for the different measurements see Table S3).

Flower color was scored by visually comparing newly opened flowers during the first week of flowering to a color chart ranging from white (saturation 0%) to pink (saturation 100%) in 5% steps using a pink color with a hue of 297° and a brightness of 100% (HSB color system, see www.colorizer.org).

Primary seed predation by *H. bicruris* larvae (i.e. seed predation resulting from oviposition and not from late instar larvae moving to a new plant) was assessed per plant by searching for characteristic entry holes and frass on developing capsules as well as by dissecting mature capsules [35], [36], [41]. Fruit maturation takes three to four weeks and primary seed predation was assessed at three to six visits for each plant, depending on the length of an individuals fruiting phase. Fruit abortion occurred only in three F_2_ individuals, and in two of these cases, signs of primary seed predation had previously been detected. All three individuals were included in the analysis.

### Scent collection

As scent emission in *S. latifolia* is quickly altered after pollination [51], we measured floral scent only on individuals that presented virgin flowers opening for the first night thereby excluding that pollination or egg laying of *H. bicruris* occurred before scent sampling. On ten days between May 12^th^ and July 22^nd^ 2008, we sampled scent for a total of six *S. dioica*, eight *S. latifolia* and 97 F_2_ hybrids (compare Table S3). The reduction in sample size for floral scent as compared to flowering, flower color and seed predation measures was caused by the availability of flowers that were just about to open on our ten scent sampling days and did not involve selecting study plants for any other reason. Note that, in comparison to other studies from lowland locations, most of our study individuals growing at 978 m a. s. l. were small and had few flowers (see results), thus, each individual presented only a few opportunities for scent sampling of newly opening flowers. We used the headspace sorption method to collect volatile organic compounds from flowers for one hour during peak scent emission time shortly after dusk (21:30–0:30). *Hadena bicruris* visitation or oviposition before scent sampling was excluded by enclosing flowers (1–3 flowers per plant) in an oven-baking bag (PET, Toppits, Germany) already during the early evening. A solid-phase thermal desorption filter (Tenax TA trap) was fitted into the oven bags and air was drawn through oven bags and filters with a vacuum pump (PAS-500 Micro Air Sampler, Spectrex) at a rate of ca.150 ml/min. Filters were sealed and transported at 4°C.

### GC-MS analyses

Filters were stored at −20°C and analyzed within three days after collection. We used a Thermal Desorption System (TDS3, Gerstel GmbH & Co. KG, Mülheim an der Ruhr, Germany) connected to a gas chromatography-mass spectrometry instrument (GC 6890, Agilent Technologies, Palo Alto, USA; MS detector 5975 A, Hewlett Packard, Atlanta, USA) fitted with an HP5 column (5%-Phenyl-methylpolysiloxane, 30 mx0.32 mm ∅x0.25 µm film thickness, Alltech, Deerfield, USA). Helium served as carrier gas. Sampled compounds were desorbed at 240°C from the Tenax TA trap with helium (99.96%) for five minutes (split-less desorption flow 8 ml min^−1^) and transferred to a cool injection system (CIS, Gerstel, at −150°C), subsequently heated at a rate of 12 C s^−1^ to 250°C with a desorption time of three minutes. Compounds were transferred via a fused silica transfer line (heated to 300°C) to the GC-MS for separation and ionization of compounds. The GC oven temperature was held at 50°C initially, increased to 250°C at a rate of 8°C min^−1^ and held at that temperature for 5 min. For all analyses, the desorption flow and column flow were kept at 8 ml^−1^ and 1.9 ml^−1^, respectively.

Authentic external standards of previously described floral scent compounds in *S. latifolia* [46] were analyzed individually by direct injection in three different amounts (1, 10, 100 ng) in the GC-MS with all parameters set as described above, to obtain calibration curves for all compounds. We obtained standard compounds from Sigma-Aldrich/Fluka (Buchs, Switzerland) with the exception of lilac aldehyde that was supplied by Stefan Dötterl (University of Bayreuth, Germany).

Chromatograms were generated with the ChemStation program (Agilent Technologies, Palo Alto, USA; 2007) and sampled compounds were identified by comparing MS spectra and retention times with those of authentic standard compounds. Absolute amounts of total floral scent and of individual compounds were calculated using peak areas of samples and calibration curves obtained for each of the analyzed compounds. Amounts of compounds were expressed as ng h^−1^ per flower. We chose to use these absolute values of scent emission for formal analysis because relative scent emission is difficult to compare between studies as scent compounds reported differ between studies. For comparison, we calculated relative contribution of each compound class to the total scent.

### Data Analysis

We first assessed the number of flowering plants as well as the incidence of primary seed predation by *Hadena bicruris* over the course of the experiment. To assess seed predation risk over time, we calculated the proportion of predated individuals for a subset of experimental plants that flowered for one week, because for these plants we know that host choice must have occurred in that week. Note that this subset of plants is not identical with the subset used for scent measurements that was determined by the availability of newly opening flowers at scent sampling days. As a second step, we compared the incidence of primary seed predation between the two species and the F_2_ individuals using a logistic regression analysis on all plants followed by pairwise proportions test with correction for multiple comparisons (see below and [52]). We describe total flower number and the length of the flowering period for comparison but did not include these data in our formal analyses (see below). This is because we did not investigate oviposition directly and do not know how many flowers were open at the time of oviposition.

We conducted two tests on flower color and floral scent measurements: (1) We tested whether previously described differences in flower color and floral scent compounds between *S. dioica* and *S. latifolia* [20], [25], were present in our field setting using exact Wilcoxon tests [53]. (2) We analyzed whether flower color and the emission of floral scent compounds likely are under additive genetic control. To do so, we used one-sample exact Wilcoxon tests [53] to test whether median trait values of the F_2_ individuals differed from the mid-parental value, estimated as the midpoint between the trait values of *S. dioica* and *S. latifolia* [54]. Corrections for multiple testing were applied across variables (see below).

Within the F_2_ plants, we further assessed whether values for flower color and floral scent compounds covered all or most of the combined range of values for *S. dioica* and *S. latifolia* as expected [54]. In addition, we tested for pair-wise correlations among flower color and floral scent compounds using Spearman rank correlations [52] with correction for multiple comparisons across correlations (see below). Correlation analyses were restricted to 91 plants used for a logistic regression model on primary seed predation such that these analyses can be compared directly (compare Table S3, see below).

The joint effect of flower color and floral scent compounds on primary seed predation by *H. bicruris* in 91 F_2_ plants (those for which all data were available, compare Table S3) was analyzed using a multiple logistic regression model [52] with primary seed predation (absent/present, see above) as the response and flower color and floral scent compounds as explanatory variables. We included the block in the experimental field as a covariate to account for spatio-temporal effects on oviposition by *H. bicruris*, compare [35]. Compounds that were emitted in less than 25% of the F_2_ individuals were not considered for this model (see results). Explanatory variables were log-transformed where appropriate (see Table S6) and all variables were centered and scaled such that effect sizes are estimated while all other variables are held at their means allowing effect size comparisons across variables [55]. We tested for full model significance using a likelihood ratio test and conservatively interpret effect estimates from the full model as this takes account of all traits jointly and avoids overestimation of effect sizes [56]. Model residuals were examined graphically and they conformed to model assumptions [52].

Corrections for multiple comparisons (see above) were applied at an overall α = 0.05 using the Benjamini-Hochberg procedure [57]. All analyses were carried out in R version 2.9.2 [58]. All data, as well as the R script used, are available freely upon request to the corresponding author.

## Results

### Flowering phenology, flower number and seed predation

*Silene dioica* flowered earlier (May 2 to May 20) than *S. latifolia* (May 27 to August 19) and F_2_ hybrids flowered across the range of both species (Fig. 1a). The number of F_2_ individuals that flowered only for one week (in total 107 of 179 F_2_ individuals with flowering status and seed predation data) peaked in late May and seed predation was substantial throughout the season (Fig. 1b). *Silene dioica* individuals (N = 33) flowered on average 2.07±0.13 weeks, *S. latifolia* individuals (N = 21) 1.33±0.14 weeks and F_2_ hybrids (N = 185) 1.50±0.05 weeks. The median total flower number per individual at the end of the season was four for *S. dioica* (range: 1–27 flowers, N = 33), three for *S. latifolia* (range: 1–14 flowers, N = 21) and four for F_2_ hybrids (range: 1–28 flowers, N = 185).

The incidence of primary seed predation per plant differed significantly between cross types (logistic regression analysis, residual deviance 281.44 on 255 df, P = 7.25 •10^−7^). Seed predation rate was very low in *S. dioica* (3.2% of the plants infested, N = 31), and differed significantly from the substantial seed predation rate in *S. latifolia* (55.6%, N = 18, X^2^ = 15.00, P = 0.0001). F_2_ hybrids also had a high seed predation rate (48.0%, N = 179) that was significantly different from that of *S. dioica* (X^2^ = 20.07, P = 7.48•10^−6^), but not from that of *S. latifolia* (X^2^ = 0.13, P = 0.718).

### Comparison of flower color and floral scent between Silene dioica and S. latifolia

Flowers of *S. latifolia* were almost invariably white (0% median color saturation) whereas *S. dioica* had a significantly higher flower color score with a median of 70% pink color saturation (Table 1, Table S4).

**Table 1:** Flower color and floral scent (ng h^−1^ per flower) and the relative contribution of compound classes to total scent (%) in field-transplanted crosses within *Silene dioica*, within *S. latifolia* and in second-generation (F_2_) hybrids between these species.

	*Silene dioica*			*S. latifolia*					F_2_ hybrids			
	median	Q_25_ a	Q_75_ a	median	Q_25_ a	Q_75_ a	*P* b ^,^ e	mid-par. Value^c^	median	Q_25_ a	Q_75_ a	*P* d ^,^ e
Flower color^f^	70	50	80	0	0	0	**1.4^.^10^−9^**	36	40	15	60	0.7914
Total scent^g^	1496	435	3461	2545	1109	4318	0.4908	1701	1061	667	2126	0.0868
**Fatty acid derivates**	**73.8%**			**51.9%**					**63.4%**			
(Z)-3-Hexenol	29.0	19.0	184.2	38.7	15.3	101.2	0.8518	31.9	27.8	5.6	116.7	**0.0029**
(Z)-3-Hexenyl acetate	377.1	108.9	2589.2	716.2	263.5	1909.3	0.9497	444.1	238.9	49.7	970.7	0.7309
Decanal	146.5	69.3	207.7	207.4	90.8	431.0	0.3450	157.9	119.7	64.5	186.6	**0.0062**
Nonanal	157.6	77.6	211.6	126.4	62.7	261.7	1.0000	138.8	132.5	69.2	205.2	0.6109
**Benzenoids**	**6.8%**			**30.5%**					**17.7%**			
Benzaldehyde	18.75	7.88	23.36	34.76	22.10	76.58	0.1079	24.02	18.56	10.70	40.00	0.7827
Benzyl alcohol	3.28	0.83	6.55	32.81	6.51	42.79	0.0813	12.98	2.85	0.40	12.70	*0.0117*
Phenylacetaldehyde	2.91	0.92	3.49	54.98	19.92	85.88	**0.0013**	20.63	4.00	1.47	23.69	0.0636
Guaiacol	0.00	0.00	0.00	0.32	0.16	1.53	**0.0037**	0.11	0.00	0.00	0.00	**0.0001**
Methyl benzoate	0.00	0.00	0.00	0.00	0.00	0.00	1.0000	0.00	0.00	0.00	0.00	0.1250
Phenylethyl alcohol	1.03	0.23	40.69	14.41	2.82	31.56	0.3280	6.25	3.31	0.32	25.52	0.0630
Veratrole	0.00	0.00	0.00	0.00	0.00	3.11	0.3287	0.00	0.00	0.00	0.00	**0.0002**
Benzyl acetate	0.00	0.00	0.00	0.00	0.00	7.33	0.4965	0.00	0.00	0.00	7.11	**4.6^.^10^−13^**
Methyl salicylate	0.06	0.01	0.54	2.87	0.37	5.35	0.0779	1.18	0.61	0.10	2.75	0.6849
Benzyl benzoate	0.00	0.00	0.27	17.59	0.00	58.17	0.1119	5.86	0.00	0.00	0.48	**4.0^.^10^−11^**
Cinnamic aldehyde	0.00	0.00	25.92	10.72	9.74	46.10	0.2151	7.06	10.46	0.00	27.12	**0.0067**
Cinnamic alcohol	0.00	0.00	32.71	24.00	19.58	58.35	0.2151	15.01	21.03	0.00	31.29	0.3343
Anisaldehyde	0.00	0.00	0.42	0.00	0.00	0.00	0.3187	0.00	0.00	0.00	0.00	**0.0020**
**Monoterpenoids**	**7.7%**			**11.3%**					**10.9%**			
α-Pinene	29.83	22.28	31.44	15.22	11.47	26.15	0.1812	21.94	20.76	11.04	40.08	0.1366
cis(Z)-Ocimene	33.28	16.78	38.60	50.43	29.27	129.26	0.4136	32.78	14.62	3.78	44.07	0.1106
Lilac aldehydes	1.66	0.000	13.68	141.18	9.95	319.22	*0.0450*	50.16	7.65	0.00	69.09	*0.0120*
Lilac alcohols	0.000	0.000	0.000	8.756	0.000	12.533	*0.0430*	2.92	0.00	0.00	0.00	**0.0008**
**Irregular terpene**	**11.7%**			**6.1%**					**6.2%**			
6-Methyl-5-hepten-2-one	127.31	79.03	190.15	150.66	47.13	230.81	0.8518	117.22	73.71	41.49	123.67	**4.7^.^10^−5^**

a quartiles (25% and 75%),

b *P*-values of exact Wilcoxon tests compare trait values between *S. dioica* and *S. latifolia*,

c mid-parental value,

d P-values of exact Wilcoxon tests comparing mid-parental trait value to F_2_ trait values,

e P-values significant after false discovery rate control at α = 0.05 using the Benjamini-Hochberg procedure are indicated are in bold type and *P*-values <0.05 in italic type,

f % pink color saturation, N = 21 for *S. dioica*, N = 16 for *S. latifolia* and N = 151 for F_2_ hybrids;

g sample sizes for scent measurements: N = 6 for *S. dioica*, N = 8 for *S. latifolia* and N = 97 for F_2_ hybrids.

During night-time floral scent collection we obtained a median of 1495 ng h^−1^ total scent compounds per flower in *S. dioica* and 2545 ng h^−1^ in *S. latifolia*; total scent emission did not differ statistically between the two species (Table 1). Overall, we identified 22 scent compounds (Table 1). Scents of both species were dominated by fatty acids (74% and 52% in *S. dioica* and *S. latifolia*, respectively), particularly by (Z)-3-hexenyl acetate. In *S. dioica*, benzenoids and monoterpenoids together constituted less than 15%, while *S. latifolia* emitted 31% benzenoids and 11% monoterpenoids (Table 1). The irregular terpene 6-methyl-5-hepten-2-one was emitted in both species in appreciable amounts (12% and 6% in *S. dioica* and *S. latifolia*, respectively). Absolute amounts of individual compounds did not differ significantly between the two species after false discovery rate control except for phenylacetaldehyde and guaiacol that were emitted in significantly higher amounts in *S. latifolia* than in *S. dioica* (Table 1). Lilac alcohols and lilac aldehydes exhibited a tendency for higher emission in *S. latifolia* than in *S. dioica* (P<0.05; Table 1).

### Trait expression and trait correlations in F_2_ hybrids

The range of trait values expressed by inter-specific F_2_ hybrids covered a large part or exceeded the combined range of *S. dioica* and *S. latifolia* for all traits, except for the benzenoids benzyl acetate and benzyl benzoate that had smaller ranges in F_2_ hybrids (Table S4). F_2_ trait values for flower color and total scent were statistically indistinguishable from the mid-parental values for these traits (Table 1). However, F_2_ medians of a number of individual scent compounds were significantly and substantially lower than mid-parental values; for example for benzyl acetate and lilac alcohols (Table 1).

Of 276 pair-wise correlations among 24 traits measured on 91 F_2_ individuals 48 were significant after correction for multiple comparisons (Table S5). Flower color was not significantly correlated with any scent compounds or with total scent. All significant correlations among individual scent compounds were positive and the strongest correlations occurred among cinnamic alcohol and cinnamic aldehyde (r_s_ = 0.94, *P*<1.0⋅ 10^−11^) as well as among the fatty acid derivates (Z)-3-hexenyl acetate and (Z)-3-hexenol (r_s_ = 0.87, *P*<1.0⋅ 10^−11^, Table S5).

### Effects of flower color and floral scent on seed predation in F_2_ hybrids

For the joint analysis of the effect of flower color and floral scent compounds on primary seed predation by *H. bicruris* we considered only the 16 scent compounds that were detected in more than 25% of the F_2_ individuals, removing the four benzenoids veratrole, methyl benzoate, guaiacol and anisaldehyde as well as lilac alcohols (compare Table S6). Of these, veratrole and lilac alcohols were emitted in low to moderate amounts in some individuals whereas methyl benzoate, guaiacol and anisaldehyde were generally emitted in trace amounts only (Table 1, Table S4).

**Figure 2:**
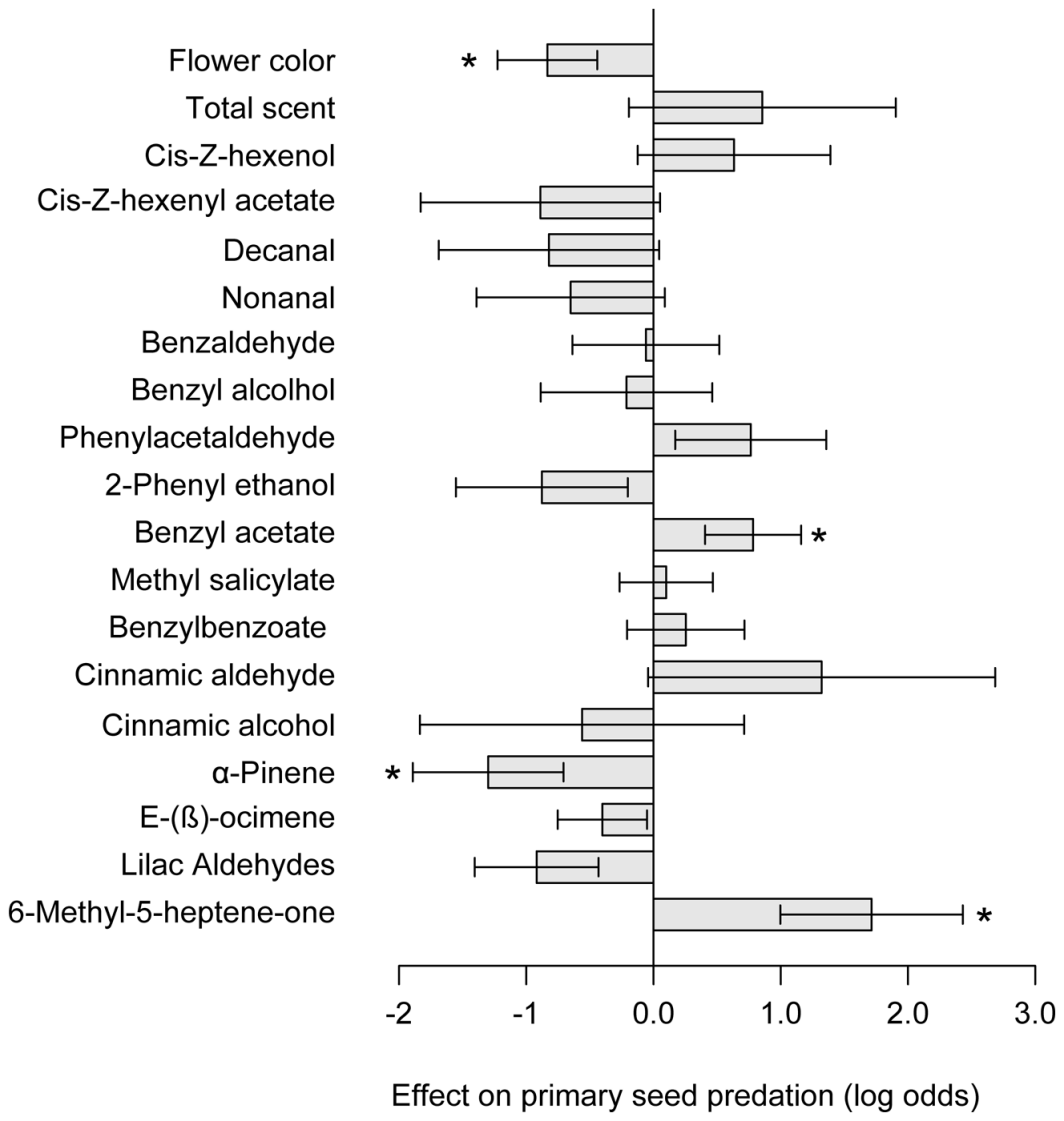
Effect of flower color and floral scent compounds on seed predation. Effect size estimates with standard errors from a logistic regression model of primary seed predation by the moth *Hadena bicruris* on inter-specific F_2_ hybrids between the moth's preferred host *Silene latifolia* and its alternative host *S. dioica*. Measurements were log-transformed where appropriate and all explanatory variables were scaled and standardized before analysis (compare Table S6). Significant effects (P<0.05) are indicated with an asterisk.

The logistic regression model on primary seed predation by *H. bicruris* in the 91 F_2_ individuals thus contained flower color, 16 scent compounds, the block effect (four blocks, i.e. three variables) and the intercept, 20 explanatory variables in total. This is an acceptable ratio of experimental units to variables of 4.55 [56]. The model had a residual deviance of 78.32 on 68 degrees of freedom indicating that the data are not over-dispersed [52] and was significant in comparison to the null model (X^2^ = 43.84, P_χ2_ = 0.0037). In this model, flower color score and emission of α-pinene significantly decreased the odds of seed predation whereas emission of benzyl acetate and 6-methyl-5-hepten-2-one increased the odds of seed predation (Fig. 2, Table S6). Note that flower color and floral scent measurements were standardized before analysis (see Material and Methods section), thus effect estimates in log odds ratio units are given for increases of one standard deviation in the trait.

## Discussion

In this study, we used field-transplanted F_2_ hybrids between *Silene latifolia* and *S. dioica* in an ecologically realistic setting to investigate the role of flower color and floral volatiles on seed predation of the *S. latifolia*-associated nursery pollinator *Hadena bicruris*. Both flower color and floral scent were related to seed predation. Pinker *S. dioica*- like flowers decreased the odds of primary seed predation suggesting that ovipositing *H. bicruris* could be attracted to the white flowers of its preferred host *S. latifolia*. Furthermore, emission of α-pinene decreased and emission of benzyl acetate and 6-methyl-5-hepten-2-one increased odds of primary seed predation. These compounds were equally present in the floral scent of the two *Silene* species in our study suggesting that they are not related to the specific interaction of *H. bicruris* with *S. latifolia*. Below we discuss the implications of these findings as well as the benefits and limitations of our approach.

*Hadena bicruris* moths are known to oviposit on a subset of flowers they visit for nectaring, and oviposition choices likely are made during nectaring and probing [31], [32], [40], [43] as in other moth species [10]. The floral traits investigated here, flower color and scent, may therefore represent visitation signals for ovipositing moths. This interpretation is, however, contingent on the assumption that flower color and floral scent are not related to hatching success and early development of *H. bicruris* larvae, i.e., before we could detect seed predation (usually when feces were visible at the outside of the capsule). Two lines of evidence suggest that this is a reasonable interpretation. First, it is clear that *H. bricruris* can develop fully on both species but attains a lower weight when feeding on *S. dioica* [33]. Secondly, studies on the interaction of *H. bicruris* with native and invasive populations of *S. latifolia* suggest that differences in oviposition rather than in hatching and larval success are crucial for realized seed predation [34], [41]. Nonetheless, plants with *H. bicruris* eggs but no larvae developing from them, possibly due to differences in plant defenses, remained undetected in our study and this could have affected our results. Moreover, selective abortion of infested fruits has been suggested as a potential defense mechanism of *S. latifolia* against seed predation by *H. bicruris* [30], [35], [36]. We followed developing fruits closely and observed fruit abortion in only three out of 91 F_2_ individuals, but this occurred only after signs of seed predation had become visible (see Materials and Methods). In contrast to *S. latifolia* individuals used for detailed studies on fruit abortion [30], [35], [36], our study plants, most of them F_2_ hybrids between *S. dioica* and *S. latifolia*, were growing at a field site at 978 m a.s.l., were small, had very few flowers and short flowering times (1–2 weeks on average). This may have contributed to the low occurrence of fruit abortion.

Our study was conducted under ecologically realistic conditions. We used *S. dioica*, *S. latifolia* and F_2_ hybrids that were transplanted into a natural *S. latifolia* population where *H. bicruris* moths were abundant. In this setting, we could not control the floral neighborhood of our study plants that can affect the behavior of by *H. bicruris* [35], [59]. Instead we used a block effect in our statistical model of primary seed predation to account spatial effects. Different from other studies on nighttime scent emission in *S. latifolia* and *S. dioica* [20], [44], [46]–[48], [51] we report floral scents that are dominated by fatty acid derivatives and contain a substantial percentage of 6-methyl-5-hepten-2-one. In studies under controlled conditions, these compounds were previously been reported as minor components of the floral scent of *S. latifolia* and *S. dioica* when detected (<3% of he total floral scent per compound [20], [47], [51]). In contrast to these studies our study plants were exposed to natural soil conditions, herbivores and competition that can dramatically impact floral and systemic scent emission [14], [16], [60]–[62]. Thus, our results advance the understanding of volatile production under field conditions that is critically needed [17].

As expected, F_2_ individuals exhibited a large variation in flower color and floral scents. However, emission of several scent compounds was significantly lower than the mid-parental value suggesting that emission of these scents may not be under full additive genetic control [54]. In addition, emission of several scent compounds was strongly correlated in the F_2_ individuals; this concerned mostly compounds produced in related biochemical pathways, for example nonanal and decanal as well as cinnamic alcohol and cinnamic aldehyde [63]. Clearly, our analysis does not allow us to fully distinguish the effects of correlated compounds. Non-additive genetic control and correlations among biochemically related compounds in F_2_ hybrids may have reduced our power to test the effects of these compounds on seed predation and are among the general limitations of using recombinant hybrids to investigate complex traits in divergent species.

F_2_ individuals with pinker *S. dioica*-like flowers were less likely to suffer from primary seed predation than those with whiter *S. latifolia*-flowers suggesting that white flower color is involved in the specificity of the *H. bicruris*-*S. latifolia* interaction. As discussed above, this could be due to differences in attracting ovipositing *H. bicruris*. Pollination by nocturnal moths, such as *H. bicruris*, is classically associated with pale or white-colored flowers [3], [4], [64] and this is supported by experiments [9], [19], [22], [23]. *Silene latifolia*-specific scents were not significantly associated with seed predation by *H. bicruris*, even though floral scent, in particular lilac aldehydes, had previously been implicated in the attraction of *H. bicruris* to *S. latifolia* [20], [44]–[46], [48]. We may have been unable to detect these effects in our study due to limited statistical power and strongly and significantly reduced emission of lilac aldehydes in the F_2_ hybrids as compared to the mid-parental value. In addition, seed predation may have been affected by ratios or combined effects of floral scents [65] or by trait correlations with unmeasured floral traits, for example nectar volume and composition, floral display and flower size [9], [10], [31], [32], [34], [66]. Nonetheless, our results are consistent with ovipositing *H. bicruris* moths relying on vision when deciding between pink-flowering *S. dioica* and their preferred host, white-flowering *S. latifolia*.

Apart from flower color, the odds of primary seed predation in F_2_ hybrids were reduced in individuals emitting larger amounts of α-pinene and increased in those emitting high levels of benzyl acetate or 6-methyl-5-hepten-2-one. These compounds are common floral volatiles [63] and have previously been reported for the floral scent of S. *latifolia* [44], [46], [47], [51], [67]. In our study, emission of benzyl acetate, α-pinene and 6-methyl-5-hepten-2-one did not differ between the two *Silene* species. Waelti *et al.* [20] also report that night-time emission of α-pinene and 6-methyl-5-hepten-2-one was similar in the two *Silene* species, but during the day, *S. dioica* emitted more 6-methyl-5-hepten-2-one and less α-pinene than did *S. latifolia* in their study. Benzyl acetate and 6-methyl-5-hepten-2-one elicit antennal responses in *H. bicruris* or other moth species [45], [68]. The compound α-pinene did not elicit antennal responses in *H. bicruris* [45] or euglossine bees [65] when tested individually. In mixtures with other scent compounds, however, α-pinene reduced the attractiveness of floral scent to euglossine bees, possibly due to receptor blocking [65], [69]. Vegetative or floral emission of all three compounds related to seed predation by *H. bicruris* in this study has been implicated in inducible reactions of plants to herbivores or pathogens: in *Sinapis alba*, floral emission of both benzyl acetate and 6-methyl-5-hepten-2-one was inhibited following aphid attack but induced in response to caterpillar feeding [70]; in wheat, barley and maize, vegetative emission of benzyl acetate was induced by *Fusarium* infection [71], [72], and herbivory caused vegetative emission of α-pinene in *Trifolium* [14] and in cotton [73]. Thus, there are two possible explanations for the associations of benzyl acetate, α-pinene and 6-methyl-5-hepten-2-one emission with seed predation: first, α-pinene could act as a deterrent and benzyl acetate and 6-methyl-5-hepten-2-one as attractants for ovipositing moths. Secondly, these compounds could be emitted as responses to herbivory or to other damage and be used by *H. bicruris* to detect high-quality *Silene* hosts. Our study provides exciting results on the relationship of benzyl acetate, α-pinene and 6-methyl-5-hepten-2-one to seed predation by *H. bicruris* on *Silene* that need to be explored further in order to understand their role in the interaction of *Silene* with *Hadena* and possibly with other biotic agents.

## Conclusions

In this field study, both flower color and floral scent of F_2_ hybrids between *Silene dioica* and *S. latifolia* were associated with seed predation by the moth *Hadena bicruris*. Our results are consistent with an important role of the white flower color of *S. latifolia* for the specific interaction with the moth *H. bircruris*. In addition, our results suggest that the incidence of seed predation is affected by the floral scent compounds α-pinene, benzyl acetate and 6-methyl-5-hepten-2-one. These compounds are not specific to either *Silene* species but have previously been implicated in induced plant reactions to herbivores and pathogens (see above). A strong influence of spatially and temporally varying agents such as herbivores or pathogens on seed predation would weaken the predictability of costs and benefits of the interaction for both partners [6], [7]. Such unpredictability would act to maintain an imperfect degree of specialization in interactions between plants and pollinating seed predators.

## Supporting Information

Table S1 **Populations.** Populations of *Silene dioica* and *S. latifolia* in the Swiss Alps that were used to generate crosses.
(XLSX)

Table S2 **Crossing design.** First- and second generation crosses within and between *Silene dioica* and *S. latifolia*, see Table S1 for population information. Family numbers in the first generation of crosses correspond to parents in the second generation. Only second-generation crosses were used in the field transplant experiment.
(XLSX)

Table S3 **Sample size information.** Families of crosses within and between *Silene dioica* and *S. latifolia* that survived in a transplant experiment, flowered and were used for measurements of flowering status, flower number, seed predation by the moth *Hadena bicruris*, flower color and floral scent. This experiment was mainly designed to obtain a highly variable hybrid population; for this reason 20 individuals of each of 36 families of second-generation (F_2_) hybrids were transplanted. 5 individuals of each of 18 families of second-generation crosses within *S. dioica* and within *S. latifolia* were transplanted for comparison. See Table S2 for crossing design.
(XLSX)

Table S4 **Ranges of flower color scores and floral scent.** Ranges of flower color and occurrence and ranges of floral scent (in ng h^−1^ per flower) in field-transplanted crosses within *Silene dioica*, within *S. latifolia* and in second-generation (F_2_) hybrids between these species.
(XLSX)

Table S5 **Correlations between flower color and floral scents.** Pair-wise Spearman rank correlation coefficients (r_s_) among flower color and floral scents in 91 field-transplanted second-generation (F_2_) hybrids between *Silene dioica* and *S. latifolia*; *P*-values significant after false discovery rate control at α = 0.05 are indicated in bold type.
(XLSX)

Table S6 **Logistic regression results.** Effect sizes with standard errors (SE) and corresponding Wald tests of floral traits and block effects in a logistic regression model of primary seed predation by the moth *Hadena bicruris* on inter-specific F_2_ hybrids between the moth's preferred host *Silene latifolia* and its alternative host *S. dioica*.
(XLSX)
